# Stimulant Treatment Gap in ADHD Patients with Heroin Use Disorder: Clinical and Behavioural Consequences

**DOI:** 10.3390/ijerph23010040

**Published:** 2025-12-28

**Authors:** Alessandro Pallucchini, Maurizio Varese, Irene Pergentini, Samuele Gemignani, Elisa Parapetto, Icro Maremmani, Angelo Giovanni Icro Maremmani

**Affiliations:** 1 Department of Psychiatry and Addictions, Section of Addictions, North-Western Tuscany Local Health Unit, Apuan Zone, Via Carriona 245, 54033 Carrara, Italy; alessandro.pallucchini@uslnordovest.toscana.it (A.P.); maurizio.varese@uslnordovest.toscana.it (M.V.); 2 Department of Psychiatry and Addictions, Section of Addictions, North-Western Tuscany Local Health Unit, Lucca Zone, Viale Marti 263, 55100 Lucca, Italy; irene.pergentini@uslnordovest.toscana.it; 3 V.P. Dole Research Group, G. De Lisio Institute of Behavioural Sciences, Via di Pratale 3, 56121 Pisa, Italy; sam.gemignani@gmail.com (S.G.); angelogiovanniicro.maremmani@unicamillus.org (A.G.I.M.); 4 Department of Psychiatry and Addictions, Section of Addictions, North-Western Tuscany Local Health Unit, Livorno Zone, Via Tiberio Scali 11, 57121 Livorno, Italy; elisa.parapetto@uslnordovest.toscana.it; 5 Saint Camillus International University of Health Sciences, Via di Sant’Alessandro 8, 00131 Rome, Italy

**Keywords:** ADHD, dual disorder, heroin use disorder, stimulant treatment, opioid agonist therapy

## Abstract

**Highlights:**

**Public health relevance—How does this work relate to a public health issue?**

- Adults with ADHD and heroin use disorder are usually treated with opioid agonist therapy, while ADHD-specific pharmacotherapy is often withheld in addiction services.
- This treatment gap affects a high-risk population with complex psychiatric needs and significant public mental health impact.

**Public health significance—Why is this work of significance to public health?**

- ADHD patients with heroin use disorder on opioid agonist therapy alone show greater emotional dysregulation, impulsivity, and cocaine use than those receiving ADHD pharmacotherapy.
- These findings indicate that current care models may insufficiently address core ADHD symptoms in dual-diagnosis patients.

**Public health implications—What are the key implications or messages for practitioners, policy makers and/or researchers in public health?**

- Integrated treatment models combining addiction care with ADHD-specific interventions should be considered for dual-diagnosis populations.
- Further longitudinal research is needed to evaluate the safety and effectiveness of combining stimulant treatment with opioid agonist therapy.

**Abstract:**

Background: Adults with attention-deficit/hyperactivity disorder (ADHD) often have comorbid substance use disorders (SUDs). In Italy, individuals with both ADHD and heroin use disorder (HUD) are usually treated in addiction services with opioid agonist therapy (OAT), but stimulant medications are rarely prescribed. This may create a treatment gap for core ADHD symptoms. Aim: This study examined the clinical and behavioural profiles of ADHD patients with HUD who receive OAT but no stimulant treatment, compared to ADHD patients without opioid use disorder (ADHD/NoHUD) on standard pharmacotherapy. All participants were considered treatment responders in their respective services. Methods: Data were collected from two outpatient clinics and included 103 adult ADHD patients assessed using validated tools for symptom severity, emotional dysregulation, and global functioning. Differences between groups were analysed using univariate tests and logistic regression. Results: The ADHD+HUD group was significantly older and showed higher levels of emotional dysregulation, impulsivity, and current cocaine use. Despite clinical stability, these individuals presented a more severe psychopathological profile than their ADHD/NoHUD counterparts, who received stimulant-based treatment. Conclusions: Although limited by its cross-sectional nature and setting-related confounders, the study indicates that OAT alone may not be sufficient to manage neurodevelopmental symptoms in ADHD+HUD patients. Further research is necessary to assess the safety and efficacy of integrated stimulant-based treatments, ideally within dual disorder services combining psychiatric and addiction expertise.

## 1. Introduction

Adults with attention-deficit/hyperactivity disorder (ADHD) often have comorbid substance use disorders (SUDs), a combination associated with increased clinical severity, poorer functioning, and difficulties in maintaining treatment engagement. A clinical question has arisen regarding whether ADHD pharmacotherapy—particularly central nervous system stimulants—can help reduce symptom burden in patients with co-occurring SUDS, including those receiving opioid agonist therapy (OAT). The current evidence on this topic remains mixed.

A recent scoping review specifically examining co-occurring ADHD and opioid use disorder (OUD) found that, although study quality and designs varied, pharmacological treatment for ADHD was generally associated with symptom improvement and, in some cases, more consistent engagement in OUD programmes. However, these associations should be interpreted cautiously, considering the observational nature of most studies and the heterogeneity of outcome measures [1]. Recent research has also highlighted the high prevalence of ADHD among patients with heroin use disorder receiving OAT, emphasising the need for improved diagnostic and treatment pathways [2].

Conversely, a clinical practice guideline based on a GRADE framework issued a strong recommendation against using psychostimulants (or atomoxetine) specifically to improve retention—although it endorsed their use (with a weak recommendation) for reducing ADHD symptoms and considered them safe within the context of SUD. This reflects the current uncertainty in the literature about whether pharmacological ADHD treatment directly aids adherence or retention in SUD care pathways [3].

Taken together, these findings suggest that stimulant-based ADHD treatment may provide symptomatic benefits for patients with OUD. However, robust evidence regarding engagement-related outcomes remains limited and inconclusive.

In fact, the literature indicates that, in real-world OUD settings, ADHD treatment—often stimulant-based—may be linked to improvements in ADHD symptom burden and treatment engagement, although high-quality evidence remains limited and inconsistent across different SUD populations. For example, more consistent benefits of stimulant treatment have been reported in ADHD patients with co-occurring stimulant use disorders, whereas findings remain mixed or inconclusive in opioid and alcohol use disorder populations, where concerns about misuse, tolerability, and limited impact on substance-related outcomes persist [4,5].

Instead of concentrating on retention, our study investigates whether the absence of ADHD pharmacotherapy in real-world OAT settings—where stimulant prescriptions are usually restricted—may be associated with different clinical and behavioural features, compared to ADHD patients treated in psychiatric settings where pharmacotherapy is standard practice.

Our research group previously examined the long-term progression of HUD patients on methadone maintenance following the Dole and Nyswander model. In that 30-year follow-up study, ADHD symptoms did not appear to influence overall clinical outcomes or the duration of treatment retention under OAT [6]. However, it remained unclear whether this treatment had any modifying effect on the underlying ADHD phenotype itself.

This raises an important and unresolved clinical question: whether the stabilising effects of OAT inadvertently mask the persistence of ADHD symptoms without effectively treating them. If so, individuals with co-occurring ADHD and heroin use disorder might continue to be under care while still experiencing untreated neurodevelopmental issues.

This study aims to explore whether the absence of ADHD-specific pharmacotherapy in patients with heroin use disorder (HUD) receiving opioid agonist treatment (OAT) is associated with distinct clinical and behavioural features, compared to ADHD patients without opioid use disorder who are on standard medication. Instead of establishing causal relationships or evaluating treatment efficacy, the goal is to generate preliminary, hypothesis-driven insights into the potential effects of this treatment disparity—particularly regarding symptom severity, emotional dysregulation, impulsivity, and the use of stimulant substances such as cocaine.

By focusing on two groups of clinically stable adult ADHD patients—one managed in addiction services with OAT alone and the other in psychiatric services with stimulant-based therapy—this study aims to describe and compare their clinical profiles to better understand the challenges and needs of dual-diagnosis populations in real-world settings.

## 2. Materials and Methods

### 2.1. Study Design and Setting

This investigation used a cross-sectional, observational design, drawing data from two separate clinical databases of specialised outpatient services in Italy: the Psychiatric Unit 2 at the University of Pisa and the Addiction Unit of the Mental Health and Addiction Department within the North-Western Tuscany Local Health Authority in Carrara. These two services represent different real-world treatment settings—psychiatric versus addiction care—each employing various therapeutic strategies for adult ADHD patients, depending on whether or not they have opioid use disorders.

In Italy, psychiatric outpatient services primarily receive referrals for adult ADHD assessment and treatment from the general mental health network and primary care, and they do not selectively admit only ‘complex’ cases. Patients with any current or past heroin use disorder are systematically excluded from ADHD pharmacological treatment in this setting and are referred to addiction services. Conversely, addiction services manage patients with heroin use disorder according to national regulatory and clinical policies. In this setting, ADHD-specific pharmacotherapy, particularly stimulant medications, is not prescribed due to safety and regulatory restrictions.

Information on previous exposure to ADHD pharmacotherapy before admission to addiction services was not systematically recorded in the retrospective databases. Consequently, it was not possible to accurately determine how many patients in the ADHD+HUD on OAT group had prior ADHD treatment or had discontinued it upon entering addiction services.

Importantly, data collection at both sites was coordinated by the same senior clinician, ensuring consistency in diagnostic procedures and data extraction.

The study included 103 adult outpatients diagnosed with attention-deficit/hyperactivity disorder (ADHD) according to DSM-5 criteria. To ensure diagnostic reliability and adequate treatment exposure, only patients who had participated in a structured therapeutic programme for at least six months, remained clinically stable, and did not discontinue treatment were included. This approach ensured some homogeneity in treatment engagement but does not allow for conclusions regarding treatment retention, as only clinically stable responders were analysed.

Participants were grouped according to clinical features and treatment environments. The first group (N = 66), called ADHD/NoHUD (ADHD without heroin use disorder), comprised individuals treated in psychiatric settings who had no current or past opioid use disorder. These patients received ADHD-specific medication, including stimulant drugs such as methylphenidate and non-stimulants like atomoxetine or bupropion, tailored individually based on their clinical response and tolerability.

The second group (N = 37), known as ADHD+HUD (ADHD with heroin use disorder), consisted of patients receiving treatment at addiction services who were on opioid agonist therapy (OAT)—such as methadone, levomethadone, or buprenorphine—but who did not receive stimulant treatment for ADHD, in accordance with current regulations and clinical practices within Italian addiction care systems.

It is important to recognise that the two groups differed not only in treatment approach but also in setting and demographic characteristics—particularly age—which may reflect different life stages and pathways into care. This naturalistic group assignment introduces potential confounding, and any differences between the groups should be interpreted cautiously and not solely attributed to treatment modality.

Clinical records were assembled by experienced psychiatrists and addiction specialists using validated tools and structured clinical interviews. Extracted variables included sociodemographic details, psychiatric history, current and previous pharmacological treatments, and patterns of substance use.

The study followed the ethical standards of the Declaration of Helsinki. All participants gave written informed consent for the anonymised use of their clinical data. Ethical approval was obtained from the Ethics Committees of the University of Pisa (Study ID: 14003, Code: ADHD-MOOD) and the North-Western Tuscany Health Authority (Study ID: 1993, Code: L-META).

### 2.2. Clinical Assessment

ADHD diagnoses were confirmed using the Italian-adapted versions of the DIVA 2.0 and DIVA-5 semi-structured interviews, which assessed all DSM-5 criteria across various developmental stages. When available, collateral informants (e.g., relatives) were involved to enhance diagnostic reliability; otherwise, diagnoses depended on patient self-report.

To evaluate ADHD symptom severity, emotional dysregulation, overall functioning, and disability, the following validated instruments were used:

- ASRS-5: A six-item self-report scale scored from 0 to 24, assessing core ADHD symptoms [7].
- RIPoSt-40: A 40-item scale measuring emotional dysregulation across four domains, with a second-order score for negative emotion regulation [8].
- CGI: A clinician-rated measure of overall illness severity [9].
- WHODAS 2.0: A 36-item assessment tool for evaluating disability across six functional domains [10].

### 2.3. Substance Use Monitoring

Substance use patterns were evaluated through a combination of structured clinical interviews and toxicological analysis of urine samples. Special focus was given to recent use of cocaine and other stimulants (excluding prescribed stimulant medications for ADHD) within the three months prior to assessment. These data were utilised to assess both current substance use and lifetime patterns, enabling group comparisons regarding substance-related behaviours.

### 2.4. Statistical Analysis

The primary analytical aim was to determine whether clinically stable ADHD patients treated in different service settings (psychiatric versus addiction care) display distinct patterns of clinical severity and behavioural features, especially focusing on the subgroup receiving opioid agonist therapy (OAT) without stimulant medication.

Statistical analyses were conducted to describe and compare the two groups—ADHD/NoHUD and ADHD+HUD—across demographic, clinical, and behavioural variables. Continuous variables were summarised as medians and interquartile ranges (Q1–Q3), while categorical variables were presented as frequencies and percentages. Group comparisons employed chi-square or Fisher’s exact tests for categorical variables and the Mann-Whitney U test for continuous variables, with statistical significance set at *p* < 0.05.

To explore potential psychological and behavioural features associated with group membership, a binary logistic regression model was used as an exploratory tool, comparing ADHD/NoHUD and ADHD+HUD participants. Predictor variables included individual items from the RIPoSt-40 and ASRS-5 scales that showed significant differences at the univariate level. A backwards elimination procedure based on likelihood ratio testing (removal criterion: *p* < 0.10) was employed. Odds ratios (ORs) with 95% confidence intervals (CIs) were reported. Analyses were carried out using IBM SPSS Statistics for Macintosh, Version 25.0.

Given the study’s cross-sectional, observational design and the absence of adjustment for key covariates, such as age or setting, the regression model’s findings should be approached with caution and viewed as hypothesis-generating rather than definitive or predictive.

## 3. Results

### 3.1. Clinical Features of the ADHD+HUD Group During OAT

Among the 103 ADHD patients, 37 individuals (36%) were receiving opioid agonist therapy (OAT) at the time of assessment, constituting the ADHD+HUD group. They had a mean age of 47 years (SD = 11.7), were predominantly male (73%), and were largely single (92%), because we defined participants in stable long-term relationships as unmarried. Educational attainment was generally low, with 75.7% having only completed lower secondary education.

Pharmacologically, 28 patients received an average daily dose of 60 mg (SD = 34) of racemic methadone, two patients were treated with 55 mg (SD = 7) of levomethadone, and seven with 8 mg (SD = 7.4) of buprenorphine. The mean duration of OAT was 4 years (SD = 5.1).

Substance use was very common: 89.2% reported current use of at least one psychoactive substance. Specifically, 29.7% continued to use opioids, 40.5% used alcohol or sedatives, and 37.8% used cannabis. Lifetime use was even higher: 83.8% for cannabis, 91.9% for cocaine or stimulants, with 56.8% reporting current use of the latter.

Psychiatric comorbidity was observed in 62.2% of cases, primarily involving mood disorders (43.2%), borderline personality disorder (16.2%), anxiety disorders (16.2%), other neurodevelopmental conditions (18.9%), and eating disorders (5.4%).

Family history data showed high rates of psychiatric disorders (73%), with reports of suicide (21.6%) and suicide attempts (40.5%) among relatives.

At the time of assessment, 73% were taking psychiatric medications: 37.8% used antidepressants, 24.3% antipsychotics, and 43.2% mood stabilisers. The most common presentation was the combined ADHD type (73%), followed by hyperactive/impulsive (16.2%) and inattentive (10.8%) subtypes.

### 3.2. Clinical Features of the ADHD/NoHUD Group During ADHD-Specific Treatment

The ADHD/NoHUD group consisted of 66 patients (64% of the sample) receiving medication specifically for ADHD. This group was significantly younger, with an average age of 25 years (SD = 9.1), and mainly male (78.8%). Educational backgrounds were more evenly distributed: 40.9% had lower secondary education, 37.9% upper secondary, and 21.2% possessed a university degree.

Pharmacological treatment included methylphenidate (N = 35; average dose: 40 mg, SD = 22.4), atomoxetine (N = 29; 40 mg, SD = 15.2), or bupropion (N = 2; 150 mg, SD = 86.6), with an average treatment duration of 1.29 years (SD = 1). Most patients were single (94%).

Substance use was still common: 72.7% reported current use of at least one psychoactive substance. Alcohol or sedative use was reported by 31.8%, cannabis by 53%, and cocaine or stimulant use by 18.2% (lifetime use: 45.5%).

Psychiatric comorbidity was found in 81.8% of the group, with mood disorders (62.1%), anxiety disorders (45.5%), borderline personality disorder (24.2%), other neurodevelopmental conditions (22.7%), and eating disorders (10.6%) being the most common.

Family history revealed high rates of psychiatric disorders (78.8%) but lower rates of suicide (1.5%) or suicide attempts (6.1%) among relatives.

At the time of assessment, 71.2% were taking psychotropic medication: 30.3% on antidepressants, 21.2% on antipsychotics, and 43.9% on mood stabilisers. The most common presentation was combined ADHD (77.3%), followed by inattentive (16.7%) and hyperactive/impulsive (4.5%) subtypes.

### 3.3. Between-Group Differences

Significant differences emerged between the ADHD/NoHUD and ADHD+HUD groups across various sociodemographic and clinical variables. Participants in the ADHD+HUD group were notably older than those in the ADHD/NoHUD group, with median ages of 47 and 25 years, respectively (*z* = −5.226, *p* < 0.001). Clinical severity, as assessed by the Clinical Global Impressions (CGI) scale, was also greater in the ADHD+HUD group (median = 4) compared to the ADHD/NoHUD group (median = 3) (*z* = −5.909, *p* < 0.001).

Regarding substance use, current consumption of cocaine or other stimulants was notably higher in the ADHD+HUD group (56.8%) compared to the ADHD/NoHUD group (18.2%) (χ^2^(1) = 16.202, *p* < 0.001).

Regarding psychiatric comorbidities, the overall prevalence was higher in the ADHD/NoHUD group (81.8%) compared to the ADHD+HUD group (62.2%) (χ^2^(1) = 4.854, *p* = 0.028). Conversely, a family history of suicide was more commonly reported in the ADHD+HUD group (21.6%) than in the ADHD/NoHUD group (1.5%) (χ^2^(1) = 12.019, *p* = 0.001).

Finally, the hyperactive/impulsive ADHD subtype was more prevalent in the ADHD+HUD group (16.2%) compared to the ADHD/NoHUD group (4.5%) (χ^2^(1) = 3.148, *p* = 0.044).

### 3.4. Clinical Characterisation of Individuals with ADHD and Heroin Use Disorder Undergoing Opioid Agonist Therapy

Table 1 summarises the results of the binary logistic regression model used to identify psychological predictors associated with membership in the ADHD+HUD group (coded as 1) versus the ADHD-OUD group (coded as 0).

Clinically, individuals in the ADHD+HUD group tend to display notable emotional lability, fluctuating between tension, irritability, and transient euphoric states in response to minor events. They often report feeling overwhelmed by everyday concerns, exhibiting pervasive anxiety and anticipatory stress, alongside a tendency to react impulsively or aggressively to frustration. Unlike ADHD patients without a history of heroin use, they appear less inclined to engage others positively, showing diminished enthusiasm in social interactions and a reduced drive to share emotional highs.

This profile shows a pattern of internalised dysregulation coupled with externalising reactivity, possibly reflecting the long-term emotional effects of unresolved ADHD symptoms managed without targeted pharmacotherapy.

## 4. Discussion

Although both groups remained clinically stable and in their respective treatment programmes, notable differences emerged in clinical presentation and patterns of substance use. Patients with ADHD and a history of heroin use disorder (ADHD+HUD), maintained on opioid agonist therapy (OAT), exhibited greater clinical severity compared to those with ADHD alone (ADHD/NoHUD), who received ADHD-specific pharmacotherapy, including stimulants.

The ADHD+HUD group was notably older, less educated, and exhibited greater overall clinical severity. They also demonstrated higher levels of emotional dysregulation, including increased mood lability, heightened frustration sensitivity, anticipatory anxiety, and exaggerated emotional reactions to minor stressors. Conversely, they reported lower scores on positive emotional engagement, indicating a reduced ability to share affective experiences.

The ADHD+HUD group, while on opioid agonist treatment (OAT), showed a higher prevalence of substance use disorders (SUDs), particularly involving stimulants like cocaine. This pattern may suggest that neither methadone nor buprenorphine fully alleviates core ADHD symptoms. One explanation is that these individuals might turn to cocaine as a form of “relief” craving—an attempt to self-regulate emotional and cognitive distress associated with untreated ADHD symptoms. Such dynamics could impair functioning in social and occupational domains, as previously documented in the literature [11,12,13].

This hypothesis is indirectly supported by the higher ASRS scores observed in the ADHD+HUD group, which may indicate a greater burden of ADHD-related symptoms. These findings could suggest the need to reconsider ADHD-specific pharmacological treatments for certain individuals with a history of heroin dependence, provided such treatments are carefully monitored.

The increased prevalence of the hyperactive/impulsive subtype in this group may indicate persistent neurodevelopmental traits that are not fully managed by OAT alone. If confirmed, this implies that ADHD symptoms require direct pharmacological intervention rather than relying on secondary improvements from opioid treatment.

On a neurobiological level, stimulant medications—especially at higher doses—have been shown to interact with the μ-opioid receptor (MOPR) system, potentially influencing receptor stability [14,15,16]. At the same time, methadone may decrease mesocortical dopaminergic activity, which could contribute to amotivation and the persistence of ADHD symptoms [17]. This complex interaction raises questions about whether current monotherapies are adequate for dual-diagnosis populations and may support the need for more nuanced, personalised strategies [18].

Data from the RIPoSt-40 further emphasise this issue: emotional dysregulation was more pronounced in the ADHD+HUD group, with elevations in emotional lability, impulsivity, and negative affect. While OAT is known to improve treatment adherence and stabilise addictive behaviour [6], it may not be sufficient to mitigate affective symptoms related to ADHD [19]. Emerging evidence suggests that methylphenidate can positively influence both ADHD core symptoms and emotional dysregulation [20,21].

Interestingly, treatment retention under OAT does not seem to differ between HUD patients with or without ADHD [6]. Methadone may help reduce impulsivity and mood swings [22], although concurrent use of sedatives or benzodiazepines—common in this group—might worsen emotional instability and reinforce unhealthy patterns of use [23,24].

Among more complex patients—such as those with underlying neurodevelopmental conditions—OAT alone may not ensure sufficient neurobiological stabilisation [25]. These observations align with a growing consensus regarding the need for precision psychiatry in addiction care [26].

In conclusion, while OAT remains essential for addressing the addictive component of dual disorder and enhances quality of life and emotional stability [27], it may largely neglect ADHD-related psychopathology [6]. This shortcoming could partly explain the higher overall clinical severity observed in ADHD+HUD patients [28].

Our findings support the hypothesis that combining ADHD-targeted stimulant pharmacotherapy with OAT could be a more effective treatment approach for selected individuals. Without such integration, the compensatory use of cocaine may indicate attempts at self-medication. Further research into the safety and efficacy of these combined treatments is necessary.

Notably, studies from Norway have already documented a growing trend in ADHD pharmacotherapy among patients receiving OAT, particularly with methylphenidate. While buprenorphine patients were more likely than those on methadone to receive ADHD medications, overall prescription rates remain low relative to the estimated prevalence of ADHD in this population [29,30]. These findings further support the hypothesis that stimulant treatment may reduce stimulant misuse (e.g., cocaine) in individuals with co-occurring heroin addiction and ADHD.

### Limitations

Several limitations must be acknowledged when interpreting these findings. Most notably, the comparison between groups is influenced by both age and treatment setting. Patients in the ADHD+HUD group were significantly older and recruited from an addiction service, whereas those in the ADHD/NoHUD group were younger and treated at a university psychiatric clinic. These differences in clinical context, care models, and patient pathways complicate any interpretation of treatment effects and limit the generalisability of between-group comparisons.

Furthermore, the study’s cross-sectional and non-randomised design prevents any conclusions about causality. All data were collected at a single point in time, making it impossible to determine whether the observed clinical differences existed before treatment, resulted from it, or reflected underlying life-stage factors.

Only patients retained in care for at least six months were included, and all were regarded as “responders” to their respective treatment programmes. Consequently, the study does not provide information about treatment retention itself—despite this being a recurring theme in the introduction and discussion—and may underrepresent more unstable or disengaged patients.

The small sample size, especially in the ADHD+HUD group, along with the number of univariate comparisons, further limits statistical power and increases the risk of type I error. The logistic regression model, although exploratory and hypothesis-generating, remains preliminary; predictors were selected based on univariate differences and were not adjusted for key covariates such as age or setting.

Substance use data were collected through self-report and toxicology screenings, but the frequency and context of use relied on subjective recall and could be affected by underreporting or social desirability bias. Additionally, the absence of longitudinal follow-up means that we are unable to comment on functional outcomes, relapse, or changes in ADHD symptoms over time.

Lastly, our findings are based on two clinical sites in Italy, each with different institutional practices and population profiles. This limits the generalisability of the results to other healthcare systems.

Despite these limitations, the study highlights a potentially important clinical gap: the unequal access to ADHD-specific pharmacotherapy among individuals with a history of heroin use disorder. Future research with larger, longitudinal samples and controlled designs is essential to assess whether combined pharmacological strategies might offer clinical benefits in complex dual-diagnosis populations [31].

## 5. Conclusions

This exploratory study highlights a potentially important gap in the treatment of individuals with co-occurring ADHD and heroin use disorder (ADHD+HUD). Although both groups studied were clinically stable and remained in care, only those without a history of opioid use received ADHD-specific pharmacotherapy. The differences observed in symptom severity and stimulant use patterns may suggest that untreated neurodevelopmental symptoms in the ADHD+HUD group may lead to more complex clinical profiles. Given the regulatory and institutional barriers that currently restrict the use of stimulants in addiction services, further research is needed to evaluate the safety, effectiveness, and long-term outcomes of ADHD pharmacotherapy—particularly stimulant treatment—in this underserved population [2]. Prospective studies employing standardised protocols and better control of confounding variables such as age and setting are required to inform evidence-based practice. Importantly, future investigations should focus on integrated care models. In this study, the two patient groups were treated within entirely separate clinical systems—psychiatric versus addiction services—with limited overlap in treatment philosophy and expertise. Dual disorder services, which combine psychiatric and addiction skills within a unified framework, may be the most appropriate setting for treating patients with complex comorbidities such as ADHD+HUD. Developing and evaluating such integrated approaches should be a priority for future research and clinical policy.

## Figures and Tables

**Table 1:** Predictors of ADHD+HUD membership based on RIPOST and ASRS scores.

	B	OR	95% CI	*p*
Reactivity, Intensity, Polarity and Stability questionnaire (RIPoSt)				
37. Emotions follow cyclical ups and downs	0.467	1.596	(1.195–2.131)	0.002
18. Feel euphoric after solving small personal problems	0.739	2.094	(1.347–3.254)	0.001
16. Eager to share excitement with others	−0.593	0.553	(0.388–0.787)	0.001
27. Worry about everything	0.466	1.594	(1.168–2.173)	0.003
03. Get nervous or react quickly when frustrated	0.726	2.068	(1.453–2.943)	<0.001
Adult ADHD Self-Report Screener (ASRS-5)				
05. Often postpone things until the last minute	0.604	1.830	(1.171–2.858)	0.008
ASRS-TOTAL-score	0.103	1.109	(1.012–1.215)	0.027

Group coding: 0 = ADHD-no HUD (patients with ADHD but no HUD), 1 = ADHD+HUD (patients with ADHD and HUD).

## Data Availability

The raw data supporting the conclusions of this article will be made available by the authors upon request.

## Abbreviations

The following abbreviations are used in this manuscript:

A-ADHD	Adult Attention-Deficit/Hyperactivity Disorder
ADHD	Attention-Deficit/Hyperactivity Disorder
ASRS-5	Adult ADHD Self-Report Scale for DSM-5
BDZ	Benzodiazepine
BMI	Body Mass Index
CGI	Clinical Global Impressions Scale
CI	Confidence Interval
ADHD-NoHUD	ADHD Patients without Opioid Use Disorder
ADHD+HUD	ADHD Patients with Heroin Use Disorder
DSM-5	Diagnostic and Statistical Manual of Mental Disorders, Fifth Edition
ED	Emotional Dysregulation
HUD	Heroin Use Disorder
MMT	Methadone Maintenance Treatment
MOPR	μ-Opioid Receptor
OAT	Opioid Agonist Therapy
OUD	Opioid Use Disorder
OR	Odds Ratio
Q1–Q3	First and Third Quartile (Interquartile Range)
RIPoSt-40	Reactivity, Intensity, Polarity and Stability Questionnaire (40-item version)
SD	Standard Deviation
SUD	Substance Use Disorder
THC	Δ9-Tetrahydrocannabinol
WHODAS 2.0	World Health Organization Disability Assessment Schedule, 2.0
